# Application of Near-Infrared Reflectance Spectroscopy for Predicting Chemical Composition of Feces in Holstein Dairy Cows and Calves

**DOI:** 10.3390/ani14010052

**Published:** 2023-12-22

**Authors:** Yiming Xu, Tianyu Chen, Hongxing Zhang, Yiliyaer Nuermaimaiti, Siyuan Zhang, Fei Wang, Jianxin Xiao, Shuai Liu, Wei Shao, Zhijun Cao, Jingjun Wang, Yong Chen

**Affiliations:** 1 College of Animal Science, Xinjiang Agricultural University, Urumqi 830052, China; xuyiming1028@163.com (Y.X.); siyuan.zhang2@uqconnect.edu.au (S.Z.); w15152786127@163.com (F.W.); dksw@xjau.edu.cn (W.S.); 2 State Key Laboratory of Animal Nutrition and Feeding, College of Animal Science and Technology, China Agricultural University, Beijing 100193, China; chentianyu@cau.edu.cn (T.C.); xingxing@cau.edu.cn (H.Z.); ylyr323@163.com (Y.N.); xiaojianxin-dairy@cau.edu.cn (J.X.); liushuaicau@cau.edu.cn (S.L.); caozhijun@cau.edu.cn (Z.C.)

**Keywords:** cattle, fecal chemical composition, calibration, near-infrared reflectance spectroscopy, modified partial least squares

## Abstract

**Simple Summary:**

Digestibility is a crucial factor for assessing feed costs for dairy cattle. Rapid analysis of fecal composition is necessary to obtain accurate data on digestive efficiency. Traditional wet chemical analysis methods are time-consuming; therefore, the potential of near-infrared reflectance spectroscopy technique was explored for assessing fecal chemical components in dairy cattle. The technique has been widely used to predict the nutritional contents of raw materials and complete feeds. In the present study, near-infrared reflectance spectroscopy could successfully predict the chemical composition of dairy cattle feces, thereby reducing analysis time and workload.

**Abstract:**

Traditional methods for determining the chemical composition of cattle feces are uneconomical. In contrast, near-infrared reflectance spectroscopy (NIRS) has emerged as a successful technique for assessing chemical compositions. Therefore, in this study, the feasibility of NIRS in terms of predicting fecal chemical composition was explored. Cattle fecal samples were subjected to chemical analysis using conventional wet chemistry techniques and a NIRS spectrometer. The resulting fecal spectra were used to construct predictive equations to estimate the chemical composition of the feces in both cows and calves. The coefficients of determination for calibration (RSQ) were employed to evaluate the calibration of the predictive equations. Calibration results for cows (dry matter [DM], RSQ = 0.98; crude protein [CP], RSQ = 0.93; ether extract [EE], RSQ = 0.91; neutral detergent fiber [NDF], RSQ = 0.82; acid detergent fiber [ADF], RSQ = 0.89; ash, RSQ = 0.84) and calves (DM, RSQ = 0.92; CP, RSQ = 0.89; EE, RSQ = 0.77; NDF, RSQ = 0.76; ADF, RSQ = 0.92; ash, RSQ = 0.97) demonstrated that NIRS is a cost-effective and efficient alternative for assessing the chemical composition of dairy cattle feces. This provides a new method for rapidly predicting fecal chemical content in cows and calves.

## 1. Introduction

Feeding practices play a pivotal role in the cost management of milk production in dairy farms [1]. Improving the digestibility of feed, aimed at minimizing feed waste, is important for reducing the cost of feeding. The assessment of feed digestibility in dairy cattle is a multifaceted endeavor that encompasses methodologies such as total fecal collection, indicator methods, nylon bag assays, and in vitro digestion tests. Regardless of the technique chosen, the determination of feed and fecal chemical composition is a critical element in the evaluation of digestibility. However, traditional wet chemistry analysis for assessing fodder nutrient digestibility is labor-intensive and time-consuming, leading to substantial inefficiency and lack of timeliness [2]. In contrast, near-infrared reflectance spectroscopy (NIRS) offers a swift and efficient alternative, enabling a timely determination process [2,3]. The application of NIRS technology has significant implications for farm management, as it reduces manual labor, costs, and waste while simultaneously enhancing profitability within the livestock sector [3]. Recent studies have demonstrated the capacity of NIRS to effectively predict the chemical composition of feeds [3,4,5,6,7,8,9].

NIRS, characterized by its cost-effectiveness and technical simplicity, possesses the capability to simultaneously measure multiple components within a sample with high precision [10,11,12]. This offers a practical and rapid alternative that does not harm the samples under examination [2]. At its core, NIRS operates on the fundamental principles involving carbon, hydrogen, and oxygen, the three elemental constituents present in all organic compounds [13,14,15]. The interaction of electromagnetic radiation with fecal dry matter compounds provides quantitative insights into the molecular composition of a sample, allowing NIRS to accurately and easily analyze the physicochemical properties of a wide array of organic samples [16].

Feces, as the end product of the intricate digestive process in the ruminant livestock gastrointestinal tract, exhibit significant variations in composition owing to differences in feed composition, intake, and fermentation processes across different rations fed to ruminants [17]. Fecal NIRS is a promising and cost-effective approach for estimating the nutritional value of diets based on fecal characteristics [10,11]. It is essential to acknowledge that the use of NIRS to predict fecal attributes is not new. Similar findings have been observed in various animal species, including cattle [6], goats [18], rabbits [19], and donkeys [20]. Nevertheless, variations in experimental results persist across different studies. The substantial compositional differences observed in the feces of cows at different growth stages necessitate the development of distinct near-infrared models [6].

The aim of this study was to rapidly predict the fecal chemical composition of dairy cattle using NIRS data, specifically focusing on parameters such as dry matter (DM), crude protein (CP), ether extract (EE), neutral detergent fiber (NDF), acid detergent fiber (ADF), ash, and starch content. Accordingly, two separate NIRS models for predicting calf and cow feces were developed.

## 2. Materials and Methods

### 2.1. Experimental Animals and Design

A comprehensive dataset consisting of 542 fecal samples was compiled and used to calibrate the NIRS model. These analyses were conducted on fecal samples collected from four separate trials conducted at different farms. Each trial measured different fecal chemical compositions, as detailed in Table 1. In pursuit of maximum variability in the dataset, fecal samples were obtained through rectal grab procedures from dairy cattle across five distinct farms situated in different regions of China, namely Beijing (116°23′ E, 39°61′ N), Baoji (107°83′ E, 34°15′ N), Daqing (124°88′ E, 47°31′ N), Harbin (126°39′ E, 45°44′ N), and Hefei (117°47′ E, 31°89′ N). These geographical variations among the farms were strategically selected to ensure a representative sample set that captured the diversity of China’s geographical environment. The cattle involved in these trials encompassed Holstein cows spanning from the post-perinatal period to 186 ± 6.88 days in milk (DIM), as well as pre-weaning calves within their initial 60 ± 4.75 days after birth. On all farms, the total mixed ration was delivered to the cows three times daily. Calves were fed whole milk twice daily. Cows and calves had access to ad libitum water. The basic characteristics of the cattle and the dietary components are detailed in Table 1.

### 2.2. Wet Chemical Analysis of Fecal Samples

Each cow received rectal stimulation from a sterile glove, facilitating the collection process three times per day for two consecutive days at 0600, 1400, and 2200 h. Approximately 300 g of feces was diligently collected at each instance. Subsequently, to preserve the integrity of the samples, 10% tartaric acid (constituting 25% of the fecal sample weight) was added, and the samples were immediately frozen at a temperature of −20 °C within sealed plastic containers [21]. These measurements were collected to maintain the samples in stable conditions until they were ready for further analysis. In the final phase, a cumulative total of 6 fecal samples were amalgamated, from which 300 g was extracted and subjected to an oven drying process at 65 °C for a duration of 48 h, followed by an additional 12 h period for moisture equilibration. The resultant dried fecal samples were processed by grinding through a 1 mm screen using a Wiley mill (Arthur H. Thomas, Philadelphia, PA, USA) and stored for subsequent testing.

Traditional wet chemical analysis methods were employed within the laboratory setting to ascertain the content of various components present in fecal samples, including DM, CP, EE, NDF, ADF, ash, and starch. To establish the DM content, air-dried samples underwent a secondary drying process at 105 °C for a duration of 4 h to attain a constant weight before analysis. Following the Association of Official Analytical Chemists (AOAC) (International, 1995), we used method 988.05 of the AOAC for CP content, method 920.39 of the AOAC for EE content, and method 924.05 of the AOAC for ash content [22]. The NDF and ADF contents were ascertained using an ANKOM 2000i automatic fiber analyzer (Beijing Anke Borui Technology Co. Ltd., Beijing, China). Finally, starch content was determined according to the methodology described by Hall [23].

### 2.3. Fecal NIRS Acquisition

Given the high sensitivity of moisture to near-infrared (NIR) radiation absorption [24], it is imperative to use dry samples to enhance the accuracy of NIRS predictions [25]. Samples were analyzed at a room temperature of approximately 25 °C. The check sample was scanned twice to complete the instrument self-test before each testing session. Each individual sample was carefully packed within a sample cup, characterized by an inner diameter of 66 mm and a height of 25 mm, and subsequently scanned across the spectral range spanning 780–2500 nm at 2 nm intervals. In this range of the spectrum, the main vibration types consist of the second (~850 nm) and third (~1000 nm) overtones of N-H, which are characteristic of proteins; the third overtone (~900 nm) of C-H, which is characteristic of lipids; and the second overtone (~950 nm) of O-H, which is typical of water [26]. The recorded data were stored as the absorbance intensity (log [1/R]) using the FOSS NIRS spectrometer DS 2500F (Foss and Infrasoft International, Silver Spring, MD, USA).

### 2.4. Preprocessing of Spectra, Calibration, and Validation

Spectral pre-treatments were implemented to mitigate the effects of instrument, sample, or environmental factors. The chemometric and spectral data outcomes were subjected to calibration and model development using WinISI III v. 1.6 (Foss and Infrasoft International, Silver Spring, MD, USA) software. A maximum standardized Mahalanobis distance (H) of 3.0 from the average spectrum was employed as a criterion to identify and eliminate outliers within the dataset. Various mathematical transformations for scatter correction and alterations in the number of modified partial least squares (MPLSs) terms were explored during the model development. Different data pre-treatment methods were assessed by varying use of scatter corrections and mathematical treatments [4,5]. Spectral data were corrected using one or more scatter corrections such as standard normal variate (SNV) and detrending (D) algorithms, multiplicative scatter correction (MSC), and/or no treatment (NO). Regression equations tested three random mathematical treatments, denoted as “1,8,8,1”, “1,4,4,1”, and “2,4,4,1”. In these expressions, the first digit represented the derivative order, with “1” indicating the first derivative of log 1/R, and “2” indicating the second derivative of log 1/R. The second digit denotes the gap between the data points for derivative calculation, whereas the third and fourth digits signify the number of data points utilized in the first and second smoothing operations, respectively.

Two distinct types of model validations were performed on the complete dataset. First, an internal cross-validation was performed using a ten-fold cross-validation procedure. During each of the ten-fold cross-validation iterations, 90% of the samples were allocated to the calibration subgroup, whereas the remaining 10% constituted the validation subgroup. The validation samples were deliberately excluded from the calibration development process to ensure the integrity of the independent validation assessment. This internal cross-validation approach prevented model overfitting by selecting the optimal number of MPLS terms for each model. Second, external validation was conducted using an independent dataset distinct from the calibration dataset, which constituted approximately one-quarter of the total dataset.

The validation samples were excluded from the calibration development process to provide a genuine opportunity for independent validation. Calibration equations were chosen using WinISI III v. 1.6 (Foss and Infrasoft International, Silver Spring, MD, USA) software based on the correlation coefficient of calibration (RSQ), correlation coefficient of cross-validation (1-VR), correlation coefficient of external validation (r^2^), root mean square error of calibration, and root mean square error of cross-validation. Additionally, the accuracy of external validation was determined based on the concordance correlation coefficient (CCC). The ratio of prediction to deviation (RPD), defined as the ratio of standard deviation to the root mean square error of prediction, was used as a measure of prediction accuracy improvement relative to the mean composition across all samples [27]. The closer the correlation coefficient value is to 1, the more accurate the model prediction results are. To evaluate the efficacy of a given model, RPD values lower than 2 were considered irrelevant for meaningful predictions [28]. Values falling within the range of 2.0 and 2.5 were considered satisfactory for qualitative feed evaluation or preliminary screening purposes. Values greater than 2.5 were regarded as acceptable for quantification. Models with an RPD value greater than 3.0 were deemed suitable for highly accurate quantitative analysis [29]. Additionally, the standard error of cross-validation (SECV) was used to establish the prediction accuracy [30].

## 3. Results

### 3.1. Sample Composition

Table 2 presents a comprehensive dataset comprising numerical data, including the number of observations, mean values, and standard deviations, all of which pertain to the chemical composition of feces calculated on a DM basis. Notably, the calibration and validation datasets demonstrated strikingly congruent ranges for each component, resulting in closely aligned mean and standard deviation values across the two datasets. This consistency underscores the robustness and reliability of calibration and validation processes.

### 3.2. Spectra Editing

**Figure 1:**
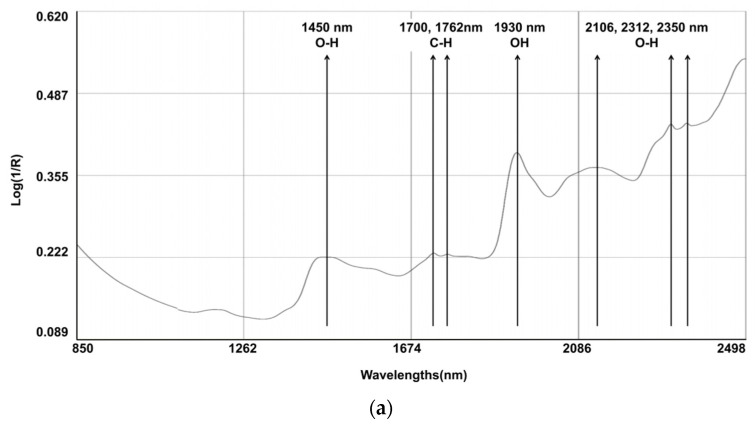

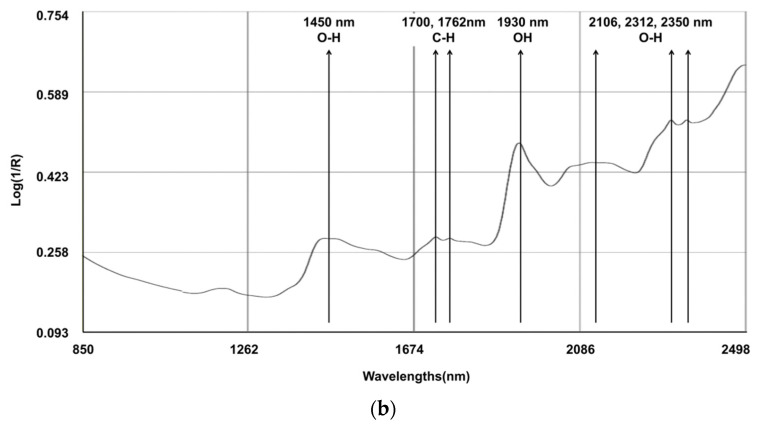
Raw average NIRS spectrum (log 1/R) for samples on (**a**) dairy cow feces (N = 329) and (**b**) calf feces (N = 213).

The spectral line depicted in Figure 1 represents the composite spectra derived from an aggregation of all the scanned samples. Several characteristic bands were identified: 1450 nm (corresponding to the O-H, which is indicative of water content); the region between 1700 and 1762 nm (associated with the C-H, indicating fat content); 1930 nm (associated with the O-H bend and indicating water); and the bands at 2106, 2312, and 2350 nm (associated with C-O, indicating the content of starch and protein). Notably, discernible variations in the reflectance spectra emerged among the fecal samples originating from different calves, particularly within the spectral range below 1700 nm. Within this segment, certain samples exhibited attenuated absorption peaks. It is pertinent to elucidate that these absorption peaks in the aforementioned spectral range are primarily attributed to the stretching vibrations of C-C, C-O, and C-N single bonds. These deviations in absorption characteristics underscore the inherent disparities in the constituents bearing these chemical bonds, which are indicative of variations in the fecal content of calves with regard to CP and NDF.

### 3.3. Calibration and Validation of MPLS Models

All samples in the dataset were used in the study, and no outliers were detected via Mahalanobis Distance. The statistical attributes of the cow equations are listed in Table 3. In the model pertaining to cows, we achieved a notably high degree of prediction accuracy for DM (RSQ = 0.98, 1-VR = 0.97) and CP (RSQ = 0.93, 1-VR = 0.91), as evidenced by RSQ and 1-VR values exceeding 0.9. The prediction models for NDF (RSQ = 0.82 and 1-VR = 0.79) and EE (RSQ = 0.91, 1-VR = 0.89) displayed slightly lower accuracies than those for DM and CP, with RSQ and 1-VR values approaching 0.9. However, less desirable outcomes were observed in the calibration equations for the prediction of ADF (RSQ = 0.89, 1-VR = 0.56), ash (RSQ = 0.84, 1-VR = 0.61), and starch (RSQ = 0.79 and 1-VR = 0.70).

The results obtained for calves differed from those observed for cows (Table 3). Among these findings, the model established for ash (RSQ = 0.97 and 1-VR = 0.96) exhibited the highest accuracy, suggesting a superior predictive performance for ash content based on all assessment criteria. The predictive models for DM (RSQ = 0.92, 1-VR = 0.86) and ADF (RSQ = 0.92, 1-VR = 0.86) displayed slightly lower accuracy than those for ash. Furthermore, for the remaining nutritional indicators, namely CP (RSQ = 0.89 and 1-VR = 0.84), NDF (RSQ = 0.76 and 1-VR = 0.55), EE (RSQ = 0.77 and 1-VR = 0.67), and starch (RSQ = 0.73 and 1-VR = 0.68), the calibration results exhibited varying levels of accuracy issues.

The results of the NIRS model for cows suggest that it provides estimates comparable to those obtained through wet chemistry for fecal DM and CP. The model yielded accurate predictions with moderate linearity for fecal EE and NDF levels. However, the results were relatively less satisfactory for other components such as ADF, ash, and starch. Conversely, for the established NIRS model for calves, the ash content was the best-predicted component, showing the potential for accurate prediction using near-infrared spectroscopy. Additionally, the DM and ADF exhibited relatively accurate calibration results, offering highly reliable predictions. However, the reliability of the predictions was lower for CP, NDF, EE, and starch.

### 3.4. Models for Accurate Determination of Compositions from Dairy Fecal Samples

**Figure 2:**
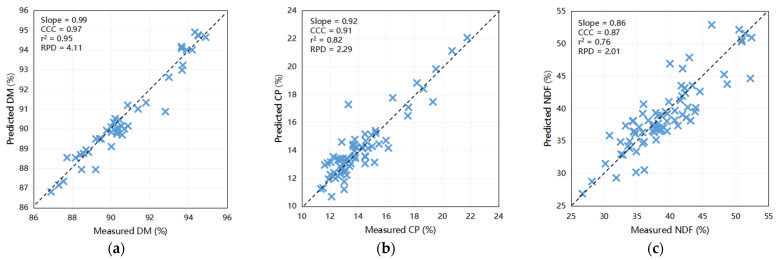

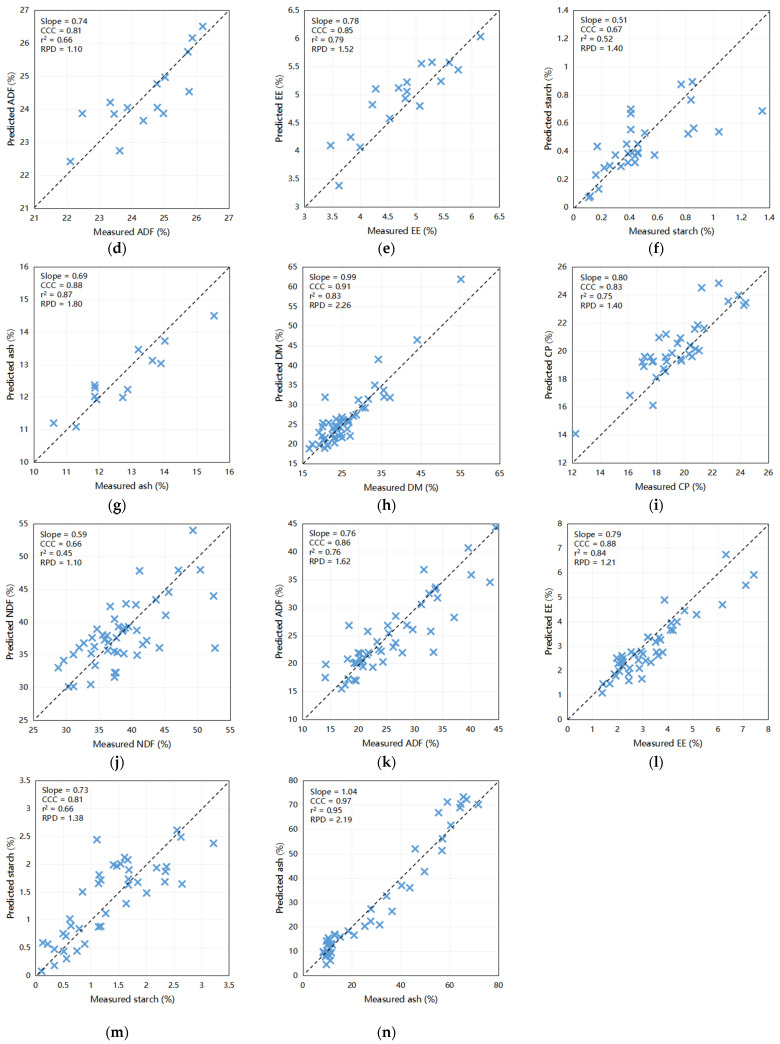
Scatter plots comparing NIRS predicted values to laboratory measured values of dairy cattle feces for various components: (**a**) DM for cows (n = 44), (**b**) CP for cows (n = 68), (**c**) NDF for cows (n = 75), (**d**) ADF for cows (n = 15), (**e**) EE for cows (n = 18), (**f**) starch for cows (n = 30), and (**g**) ash for cows (n = 13); (**h**) DM for calves (n = 51), (**i**) CP for calves (n = 35), (**j**) NDF for calves (n = 48), (**k**) ADF for calves (n = 47), (**l**) EE for calves (n = 44), (**m**) starch for calves (n = 41), and (**n**) ash for calves (n = 44). DM = dry matter; CP = crude protein; EE = ether extract; NDF = neutral detergent fiber; ADF = acid detergent fiber; r^2^ = the correlation coefficient of external validation; CCC = concordance correlation coefficient; SEP = standard error of prediction; RPD = ratio of performance to deviation (SD/SEP). Bisecting line indicate (x = y).

The outcomes of the predictive modeling of the fecal chemical composition of nutrients using an external dataset are illustrated in Figure 2. The models exhibited highest precision in forecasting DM (Slope = 0.99, CCC = 0.97, Coefficient of Determination [r^2^] = 0.95, RPD = 4.11) and CP (Slope = 0.92, CCC = 0.91, r^2^ = 0.82, RPD = 2.29) when compared to other constituents. Moreover, scatter plots depicting the relationship between NIRS and wet chemistry analysis for NDF (slope = 0.86, CCC = 0.87, r^2^ = 0.76, and RPD = 2.01), ash (slope = 0.69, CCC = 0.88, r^2^ = 0.87, and RPD = 1.80), and EE (slope = 0.78, CCC = 0.85, r^2^ = 0.79, and RPD = 1.52) yielded relatively favorable results. These findings indicate the capability of these calibration models to accurately predict the DM and CP levels in fecal samples. Furthermore, they have the potential to be exceedingly valuable for NDF, ash, and EE for the efficient prediction of large sample sizes within a short timeframe. Notably, the predictive accuracies for ADF (slope = 0.74, CCC = 0.81, r^2^ = 0.66, and RPD = 1.10) and starch (slope = 0.51, CCC = 0.67, r^2^ = 0.52, and RPD = 1.40) were comparatively lower, underscoring the need for further refinement.

The external validation results for the calf model were concordant with the internal validation results, as shown in Figure 2. Predictive accuracy was highest for ash (Slope = 1.04, CCC = 0.97, r^2^ = 0.95, RPD = 2.19) and DM (Slope = 0.99, CCC = 0.91, r^2^ = 0.83, RPD = 2.26), whereas some disparities were observed in the predictions for CP (Slope = 0.80, CCC = 0.83, r^2^ = 0.75, RPD = 1.40), ADF (Slope = 0.76, CCC = 0.86, r^2^ = 0.76, RPD = 1.62), and EE (Slope = 0.79, CCC = 0.88, r^2^ = 0.84, RPD = 1.21) when compared to the wet chemistry method. However, the predictions for starch (slope = 0.73, CCC = 0.81, r^2^ = 0.66, and RPD = 1.38) and NDF (slope = 0.59, CCC = 0.66, r^2^ = 0.45, and RPD = 1.10) did not achieve the desired levels of accuracy. These results provide valuable insights into the robustness and limitations of predictive models and suggest avenues for future refinement and enhancement.

## 4. Discussion

The fecal chemical concentrations in our study are close to the results of other studies; the samples are highly representative and can be used for modeling purposes [31,32]. The conventional approach for NIRS calibration involves the acquisition of spectral data and corresponding reference measurements from a substantial number of samples, collectively forming a calibration dataset. Subsequently, this calibration dataset was employed to formulate a predictive model capable of estimating reference values based on spectral data.

Cattle fecal compounds are composed of various chemical bonds and functional groups, including C-H, O-H, and N-H, which originate from undigested residues and end products. These components contribute to the NIR spectral information, which is closely associated with dietary digestibility. The log (1/R) reflectance spectra of the fecal samples were similar to those observed in other studies [6,33,34]. Several characteristic bands were identified: 1450 nm (corresponding to the 1st overtone of the O-H stretch, indicating water content); between 1700 and 1762 nm (associated with the 1st overtone of C-H stretch in CH_2_ and CH_3_ groups, indicating fat content); 1930 nm (a combination band of the O-H bend and stretching vibrations of water); and 2106 nm, 2312 nm, and 2350 nm (combination bands associated with C-O stretching and bending vibrations, indicating the presence of starch and protein absorption) [33].

While Brogna et al. [27] reported relatively poor accuracy for the calibration equation of the DM (RSQ = 0.77, 1-VR = 0.65, and RPD = 1.69), the current study observed a stronger performance for both DM equations. Near-infrared reflectance spectroscopy has been employed to predict the nitrogen and carbon fractions in dairy cow feces, demonstrating a robust association between wet chemistry analysis and NIRS prediction for CP (RSQ = 0.97) [35]. Other studies have also shown comparable statistical values when comparing the reference analysis and NIRS method, such as CP for dairy manure (RSQ = 0.92) [36]. Similarly, NIRS was utilized to forecast CP in beef cattle fecal samples within the wavelength range of 400–2500 nm, using partial least squares (PLS) regression. The RSQ and SECV values for CP were as follows: CP (RSQ = 0.80, SECV = 0.74) [37]. Furthermore, a calibration model was developed using NIRS to estimate pig fecal chemical composition, which performed well for CP (RSQ = 0.89, standard error of calibration (SEC) = 18.1, and SECV = 18.8) [38]. In the external validation, the correlation coefficient value for CP between the NIRS analysis and chemistry methods was 0.97, with a slope of 1.000 [35]. In this study, the NIRS calibration equation for CP in cows demonstrated higher accuracy and outperformed the predictive performance for calves.

Calibration for the determination of cow fecal NDF and ADF exhibited a relatively strong linearity in the relationship between the predicted and reference values [39]. However, the calibration model results from another experiment were less accurate, with RSQ values and SECV values as follows: N 0.80 (0.74), ADF 0.92 (12.04), and NDF 0.86 (13.50) [37]. The model for pig feces performed well for NDF (RSQ = 0.94, SEC = 55.0, SECV = 60.2) [36]. For external validation, the predictions for ADF (r^2^ = 0.85, slope = 1.10) and NDF (r^2^ = 0.74, slope = 1.03) showed intermediate accuracy with good predictions [37]. In our study, the predictions for NDF and ADF exhibited an intermediate level of accuracy.

Studies on the ash content have yielded different results. Reflectance spectra were measured within the wavelength range of 1100–2498 nm using a benchtop NIRS instrument working in reflectance mode, with prediction performances for ash (RSQ = 0.65) lower than those obtained in our study [26]. However, studies have indicated that in NIRS models for beef cattle feces under grazing conditions, predictions for ash have a high level of reliability [39]. Additionally, a model used for quantitative prediction of the ash components of wet Miscanthus samples demonstrated moderate RSQ values (0.82) when validated against a validation set [40]. Similarly, our calibration equation yielded significantly different results for the cows and calves. The calibration equation for cows had lower accuracy, but that for calves had high accuracy. The main disadvantage of NIRS is its weak sensitivity to minor constituents, such as ash [26]; thus, more accurate NIRS prediction equations require a larger reference dataset for cow fecal ash components [41].

One study found that the majority of MPLS calibrations demonstrated a strong correlation between the predicted and actual values for most components, with RSQ values exceeding 0.90, except for starch (RSQ = 0.66) [27]. However, this does not imply that all predictive models established in the experiments cannot achieve a good predictive performance, as some studies have already established reliable calibrations for fecal starch (Root Mean Square Error (RMSE) = 0.83, r^2^ = 0.88) [42]. Numerous feed laboratories in the United States currently offer NIRS analysis of fecal starch to their professional customers [27]. In our study, neither the models for cows nor calves provided accurate predictions for starch. The reason for these results may be errors in determining the wet chemistry of starch. NIRS analysis relies fundamentally on the wet chemistry analysis of calibration samples. It is crucial to emphasize that the accuracy and precision of NIRS predictions are intricately linked to the quality of wet chemistry analysis [43]. The reliability of NIRS predictions for fecal starch may be related to the variability of starch in wet chemical analysis.

There are many studies utilizing NIRS to predict the components of DM, CP, NDF, ADF, and starch in cow feces, whereas research on EE and ash is relatively limited. Relevant studies have conducted analyses of the contents of various chemical components in feed and pig feces. Parrini et al. [44] conducted a study examining the application of NIRS to determine the nutritional value and chemical composition of natural pastures, including the predictive accuracy for EE (RSQ = 0.99, 1-VR = 0.98, and r^2^ = 0.98), which showed a high level of reliability. In contrast, a study conducted by Thomson et al. [4], which evaluated the precision of NIRS analysis for grass–clover mixture silages, revealed that the 15 chemical components assessed, including EE, were found to be unsatisfactory (RSQ = 0.25). Moreover, calibration and validation equations were developed for pig feces, with the lowest results observed for fat (RSQ = 0.69, SEC = 11.7, and SECV = 12.3) compared to the other components [38]. In this study, the NIRS model for EE in cows was slightly better than those reported in other studies; however, NIRS could not accurately predict the chemical composition of feces for EE. This difference can result from a variety of factors, such as the composition of fecal EE in calves, which is more different than that in cows. However, these problems can be avoided by updating, expanding, and improving the initially developed and validated calibrations to further improve the robustness of current calibration models [14].

The study results highlight the possibility of precisely forecasting the fecal chemical structure using NIRS, and the findings hold substantial practical importance. Conducting laboratory analyses requires substantial investments in time and financial resources to collect and process samples [2]. Waiting for results consumes valuable time, and delayed results could make the data outdated for timely managerial decisions. Utilizing NIRS for rapid parameter determination has the potential to improve nutrition and health management in commercial feedlots by saving time on wet chemistry analyses and predicting feedlot diet digestibility [3]. Additionally, NIRS has been acknowledged as a cost-effective and environmentally friendly method in the field of fecal composition as it eliminates the need for chemicals and minimizes hazardous waste production [3]. These outcomes benefit feedlot owners and the environment.

The findings, although derived from a limited number of reference samples, represent a promising initial step toward the practical application of this methodology. Therefore, to obtain more reliable conclusions in the future, further research that includes a greater number of samples, animals, and farms is required [38]. This enhancement process may involve updates, expansions, and improvements to the initially developed and validated calibrations [45]. These aspects are crucial for effectively implementing accurate predictions of fecal composition, as demonstrated by the findings of this study.

## 5. Conclusions

The empirical evidence presented herein attests to the robustness and dependability of the NIRS methodology in forecasting the content of DM and CP in cow fecal matter, as well as the content of DM and ADF in calf fecal matter. Moreover, NIRS is reliable for predicting the NDF, EE, and ash content in cow fecal samples. The precision of the estimation was constrained when tasked with predicting ADF and starch content in cow feces and CP, NDF, EE, and starch content in calf feces. Consequently, the application of NIRS holds significant promise in expediting the analysis of fecal composition, thereby presenting an opportunity to supplement labor-intensive and costly wet chemistry-based laboratory methods.

## Figures and Tables

**Table 1:** Description of four trials conducted with dairy cattle from four periods at five different farms.

Trial	1				2	3		4
N ^1^	40	13	55	36	50	68	67	213
Location	Beijing	Baoji, Shaanxi	Daqing, Heilongjiang	Harbin, Heilongjiang	Beijing	Hefei, Anhui	Hefei, Anhui	Baoji, Shaanxi
Period	Peak lactating	Peak lactating	Peak lactating	Peak lactating	Mid lactating	Fresh	Peak lactating	Pre-weaning calf stage
Body weight, kg	647.50	617.20	719.30	734.50	675.50	687.70	679.40	80.70
Milk yield, kg/d	41.10	46.30	48.20	51.10	36.30	32.60	38.20	-
Fecal chemical composition ^2^	DM, CP, NDF, Starch				DM, CP, EE, NDF, ADF, Ash	DM, CP, EE, NDF, ADF, Ash		DM, CP, EE, NDF, ADF, Starch, Ash
Dietary components, % of DM unless otherwise noted								
DM, %	49.05	50.67	47.54	50.83	50.71	46.16	47.28	14.67
CP	16.71	19.43	18.08	18.79	18.80	16.64	17.17	22.30
NDF	26.21	27.22	23.72	32.94	49.27	32.71	31.05	-
EE	3.17	3.56	3.21	4.81	2.62	4.27	4.64	18.03
Starch	16.17	16.86	17.94	18.15	13.57	25.95	29.38	-
Ash	7.89	6.21	6.14	7.73	7.44	8.49	7.83	-

^1^ N = number of animals sampled for feces; ^2^ DM = dry matter; CP = crude protein; EE = ether extract; NDF = neutral detergent fiber; ADF = acid detergent fiber.

**Table 2:** Descriptive calibration data set and validation data set statistics for NIRS of fecal chemical composition.

Group	Chemical Composition ^1^	Calibration Data Set			Validation Data Set		
		*n* ^2^	Mean	SD ^3^	*n* ^2^	Mean	SD ^3^
Cows	DM (%)	128	16.86	1.33	44	16.80	1.12
	CP (% of DM)	197	14.08	2.60	68	14.15	2.21
	NDF (% of DM)	218	38.43	5.98	75	39.13	5.59
	ADF (% of DM)	39	23.99	1.83	15	24.63	1.85
	EE (% of DM)	54	4.84	1.05	18	4.75	0.75
	Starch (% of DM)	92	0.35	0.23	30	0.49	0.29
	Ash (% of DM)	41	12.09	0.91	13	12.72	1.33
Calves	DM (%)	145	25.18	6.88	51	25.82	7.49
	CP (% of DM)	99	19.99	2.45	35	19.39	2.29
	NDF (% of DM)	135	38.26	6.46	48	38.65	4.99
	ADF (% of DM)	128	24.44	8.02	47	25.85	6.66
	EE (% of DM)	128	2.65	1.46	44	3.18	1.13
	Starch (% of DM)	119	1.31	0.82	41	1.38	0.71
	Ash (% of DM)	124	18.90	22.07	44	28.90	28.20

^1^ DM = dry matter; CP = crude protein; EE = ether extract; NDF = neutral detergent fiber; ADF = acid detergent fiber; ^2^ *n* = number of sample analyses; ^3^ SD = standard deviations.

**Table 3:** Calibration and cross-validation statistics of the fecal NIRS equations to predict chemical composition (% of DM).

Group	Chemical Composition ^1^	Math Treatment ^2^	Scatter Correction ^3^	Calibration Statistics ^4^		Cross-Validation Statistics ^5^	
				SEC	RSQ	SECV	1-VR
Cows	DM	1,8,8,1	D	0.34	0.98	0.39	0.97
	CP	1,4,4,1	SNV	0.69	0.93	0.78	0.91
	NDF	1,8,8,1	SNV	2.44	0.82	2.60	0.79
	ADF	1,4,4,1	SNV + D	0.89	0.74	1.21	0.56
	EE	1,4,4,1	MSC	0.26	0.91	0.29	0.89
	Starch	1,4,4,1	D	0.10	0.79	0.12	0.70
	Ash	1,4,4,1	NO	0.37	0.84	0.57	0.61
Calves	DM	2,4,4,1	SNV + D	1.86	0.92	2.38	0.86
	CP	1,8,8,1	SNV + D	0.82	0.89	0.98	0.84
	NDF	2,4,4,1	SNV + D	3.01	0.76	4.12	0.55
	ADF	1,4,4,1	SNV + D	2.21	0.92	3.05	0.86
	EE	1,4,4,1	D	0.42	0.77	0.51	0.67
	Starch	2,4,4,1	SNV + D	0.43	0.73	0.47	0.68
	Ash	1,4,4,1	SNV + D	2.51	0.97	3.19	0.96

^1^ DM = dry matter; CP = crude protein; EE = ether extract; NDF = neutral detergent fiber; ADF = acid detergent fiber; ^2^ The digit represents, respectively, derivative order, subtraction gap, first smoothing, and second smoothing; ^3^ SNV = standard normal variate; D = detrend; MSC = multiple scatter correction; ^4^ SEC = SE of calibration; RSQ = the correlation coefficient of calibration; ^5^ SECV = SE of cross-validation; 1-VR = the correlation coefficient of cross-validation.

## Data Availability

All the data are available in the manuscript.
